# Experimental Study of Fully Passive, Fully Active, and Active–Passive Upper-Limb Exoskeleton Efficiency: An Assessment of Lifting Tasks

**DOI:** 10.3390/s24010063

**Published:** 2023-12-22

**Authors:** Ali Nasr, Clark R. Dickerson, John McPhee

**Affiliations:** 1 Department of Systems Design Engineering, University of Waterloo, Waterloo, ON N2L 3G1, Canada; mcphee@uwaterloo.ca; 2 Department of Kinesiology and Health Sciences, University of Waterloo, Waterloo, ON N2L 3G1, Canada; cdickers@uwaterloo.ca

**Keywords:** exoskeletons, wearable robots, active–passive, electromyography, sEMG, fatigue

## Abstract

Recently, robotic exoskeletons are gaining attention for assisting industrial workers. The exoskeleton power source ranges from fully passive (FP) to fully active (FA), or a mixture of both. The objective of this experimental study was to assess the efficiency of a new active–passive (AP) shoulder exoskeleton using statistical analyses of 11 quantitative measures from surface electromyography (sEMG) and kinematic data and a user survey for weight lifting tasks. Two groups of females and males lifted heavy kettlebells, while a shoulder exoskeleton helped them in modes of fully passive (FP), fully active (FA), and active–passive (AP). The AP exoskeleton outperformed the FP and FA exoskeletons because the participants could hold the weighted object for nearly twice as long before fatigue occurred. Future developments should concentrate on developing sex-specific controllers as well as on better-fitting wearable devices for women.

## 1. Introduction

Workers in Canada frequently get musculoskeletal diseases (MSDs) from their jobs, often in their upper extremities, notably, their shoulders [1]. One recent solution is the exoskeleton, which is a wearable device that can augment the wearer’s natural physical abilities [2]. Robotic exoskeletons allow the user to carry heavier objects or mitigate physical limitations [3]. Exoskeletons for the industrial workforce are being created, researched, and used more often on a global scale [4]. Exoskeleton design may be divided into three groups based on the source of assistance: FP [5], FA [2], and AP [6].

Prior to practical exoskeleton deployment across diverse work situations, several technical, physical, and psychological factors require evaluation. Analysis of these factors is not standardized and varies according to application domain, focused research objectives, and types of studies performed (simulation [7] or experiment [8]). For example, Hodson [9], Kim et al. [10] and Alemi et al. [11] have used maximum voluntary isometric contractions (MVICs) [12] for exoskeleton evaluation. The other evidence-based metrics are muscle activity measured through surface electromyography (sEMG) [13,14,15,16], fatigue/endurance using mean power frequency [16,17,18,19], muscle metabolic energy expenditure (MMEE) [11], task completion or time [10,20], subjective feedback [10,20], or discomfort feedback [11,20,21]. However, to the best of our knowledge, there have been limited efforts to employ an inverse dynamic skeletal model integrated with machine learning-based muscle models that exhibit kinematic closed-loop interactions with exoskeletons. Specifically, some prior studies have relied on simplifications in terms of the human-exoskeleton kinematic closed-loop interaction and have employed different (e.g., Hill-type) muscle models. For example, Marinou et al. [22], Sharafi and Uchida [23], and Shushtari et al. [24] conducted simulation-based evaluations of the human skeletal system with an exoskeleton (without experimental evaluation), and Kuo et al. [7] and Li et al. [25] carried out experimental evaluations using detailed Hill-type muscle models within musculoskeletal models and simplified exoskeletons without closed-loop interaction.

Despite the numerous distinctions between male and female functional and static anthropometrics [26], generally, sex has been disregarded when analyzing exoskeletons [9], or the tests had male-dominated samples [10]. The majority of industrial and physically demanding jobs are held by males, which may explain the male predominance in samples. Nevertheless, exoskeleton sizes and designs may be suboptimal for female anatomy [21]. Only a few investigations report on how exoskeleton use affected muscle activation according to sex [9,11,20,27]. Females had remarkably more median sEMG at the right triceps brachii with the exoskeleton compared to those without exoskeleton and males with the exoskeleton [20]. A few researchers showed that the MVICs are inconsistent for each muscle and for each sex, which should be added in sex-specific exoskeleton design and control [11,27]. Another difference is the time discrepancies in the donning and taking off of the exoskeleton by sex, with women taking much less time than men [28]. Kim et al. [10] anticipated that sex will cause variations in exoskeleton effectiveness evaluation. Further research is needed to characterize the interaction of sex and exoskeleton use [4,10]. Furthermore, there is an increased frequency of discourse regarding sex-based variations in body morphology and their correlation with exoskeleton fitting, as documented in the study by Sposito et al. [29].

In the context of exoskeletons, efficiency refers to the optimal utilization of energy and resources by the wearable robotic system to assist and enhance human movement. This encompasses factors such as biomechanical assistance, metabolic expenditure reduction, and mechanical advantage, all of which contribute to the overall effectiveness and performance of the exoskeleton in aiding the wearer’s mobility and physical tasks. The study investigates how these efficiency metrics are influenced by sex differences, shedding light on potential variations in exoskeleton performance between male and female users.

The main goal of this study was to discover the relative efficiency of wearable exoskeleton devices with regard to sex. Specifically:

I.  Comparing the efficiency of FP, FA, and AP shoulder exoskeleton in human-in-the-loop (HITL) experiments;
II.  Evaluating with 12 criteria within categories of (1) sEMG channels, (2) kinematic data, and (3) survey;
III. Reporting and assessing the influence of sex on these criteria.

First, the experimental setup and test protocol are introduced in Section 2. Second, the evaluation metrics are developed and discussed in Section 3 using independent sub-component models. Finally, the results for different criteria and sex are presented and discussed in Section 4.

## 2. Methodology

**Figure 1:**
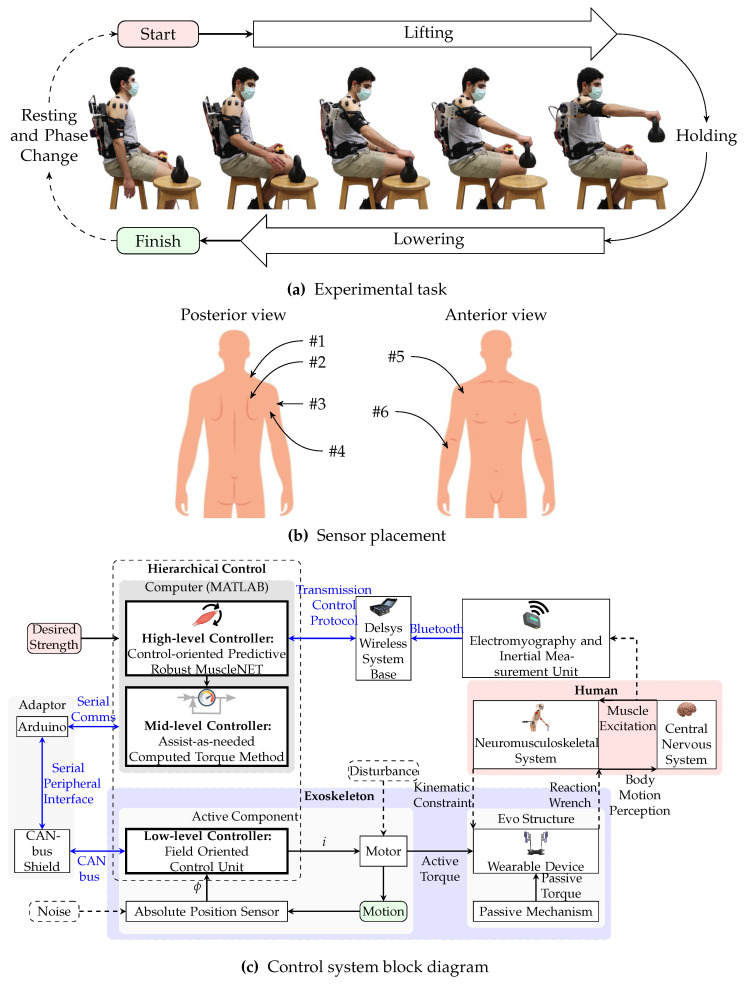
(**a**) The subject is doing a weight-lifting exercise in the sagittal plane while wearing the exoskeleton and sensors; (**b**) the placement of wireless sEMG-IMU sensors to the skin at sites of #1 UTRA, #2 MTRA, #3 MDEL, #4 PDEL, #5 ADEL, and #6 BRD located on the right forearm, shoulder, and upper trunk musculature; and (**c**) block diagram showcasing the primary components of the control system and the connection protocols utilized [8].

To evaluate the assistance provided by the exoskeletons, twenty healthy participants lifted an object (5lb kettlebell) and held to exhaustion while wearing the exoskeleton (Figure 1a) as the sEMG sensors measured their muscle activity. They repeated the test with different exoskeleton support modes until fatigue occurred. While the object’s weight may fall within the lighter range of loads encountered in certain industries, it symbolizes the sustained nature of tasks that workers often face. By examining the exoskeleton’s performance under such conditions, we aim to shed light on its potential to alleviate fatigue and enhance endurance, which are vital considerations for preventing workplace injuries and improving overall efficiency in various occupational settings.

Twenty participants (mean ± Standard deviation (STD): 25±3.3years; 64±12.9kg mass; 1.74 ± 0.09 m height; 2.0±1.8 workout sessions per week; 18 right-handed and 2 left-handed) participated. Ten of the participants were female (mean ± STD: 24±3.0years; 59±10.6kg mass; 1.65±0.07m height; 3.0±1.9 workout sessions per week; 9 right-handed and 1 left-handed) and the rest were male (mean ± STD: 27±3.1years; 74±11.7kg mass; 1.78±0.06m height; 1.0±1.7 workout sessions per week; 9 right-handed and 1 left-handed). Participants were between the ages of 20 and 50 and without a recent injury or history of discomfort in their upper extremities. Each participant signed a written waiver of informed consent. The University of Waterloo’s Office of Research Ethics approved the experimental protocol.

Participants completed activities according to Table 1. In the first phase, the participant performed MVIC tasks for normalizing the sEMG signals and calibrating the brushless direct current (BLDC) motor home angle. Participants performed free motion with a weight during the second phase. The information from this phase was used to generate training data for the machine learning mapping electromyography to kinematic and dynamic biomechanical variables (MuscleNET) model [30]. To evaluate the various modes of support (inactive exoskeleton (IE), FP, FA, and AP assistance), the participant repeated the weight-lifting task for each support mode. The participant was instructed to maintain the kettlebell at a $90^{\circ }$ elevation angle (Figure 1a); however, precise control of the elevation angle was not enforced, relying instead on vocal feedback provided by the researcher. Furthermore, there were no specific constraints imposed regarding movement velocity, and participants were encouraged to lift the object at a comfortable pace. Following each repeated task, participants were mandated to observe a minimum resting interval of 10 min to mitigate the onset of fatigue.

sEMG sensors and a custom adapted commercial shoulder exoskeleton were the two main devices used:

I.  The Delsys Trigno wireless compact system (Delsys Inc., Natick, MA, USA) is equipped with two integrated sensors designed for the measurement of sEMG to assess muscle activity and an inertial measurement unit (IMU) to capture kinematic data, including Euler angles and angular acceleration. To collect data, these wireless compact units were affixed to the skin at six specific anatomical sites (Figure 1b): #1 upper trapezius (UTRA), #2 middle trapezius (MTRA), #3 middle deltoid (MDEL), #4 posterior deltoid (PDEL), #5 anterior deltoid (ADEL), and #6 brachioradialis (BRD) located on the right forearm, shoulder, and upper trunk musculature. The data obtained from these sensors, encompassing both surface muscle activity and Euler angles, were utilized as inputs for the MuscleNET framework, with further details about MuscleNET available in reference [8].
II. Motorized EVO (Ekso Bionics Holdings Inc., San Rafael, CA, USA) upper limb exoskeleton with built-in three-level passive assistance was used to assist the shoulder elevation joint. The motor was an AK80-9 KV100 BLDC motor (Cubemars, Jiangxi Xintuo Enterprise Co., Nanchang, China) with a built-in relative encoder, 0.485kg mass, 9Nm rated torque, and 9:1 gear ratio, which was used for the active component. The exoskeleton was optimally designed in [6] after being modeled, including its passive torque-angle function. The active assistance (motor) is controlled with a hierarchical control structure (Figure 1c) that used a subject-specific MuscleNET-driven intention prediction model [8,31].

The exoskeleton configuration encompasses four distinct modes, each serving a specific purpose in the augmentation of human movement:

- Inactive exoskeleton (IE) setting: In this mode, the exoskeleton remains dormant, providing no assistance to the user. This setting serves as a baseline for evaluating the unaided human performance during the task.
- Fully passive (FP) setting: The exoskeleton operates in a passive manner, employing a spring mechanism to generate assistive torque in correlation with the angle of the user’s shoulder elevation. This assists the wearer in counteracting the gravitational forces acting on the lifted object.
- Fully active (FA) setting: Activating the lightweight BLDC motor, this mode delivers targeted assistance based on the user’s muscle contribution and intent. The motor’s engagement is calibrated to provide a measured level of support, enhancing the user’s lifting capability.
- Active–passive (AP) setting: This setting synergistically combines both the passive spring mechanism and the active BLDC motor to jointly deliver assistive torque throughout the user’s exoskeleton elevation angle. The collaboration between these elements aims to optimize the wearer’s performance by harmonizing mechanical support and motorized assistance.

## 3. Evaluation Criteria

The utilization of multiple criteria for assessing exoskeleton performance is crucial for providing a comprehensive and holistic understanding of its effectiveness. Relying on a single criterion might oversimplify the evaluation process and potentially overlook nuanced insights into their performance across different contexts and functionalities. By incorporating a diverse set of criteria, our study aims to capture the multifaceted nature of exoskeleton efficiency, enabling a more nuanced analysis that accounts for a range of factors influencing their overall performance and impact on users. This approach enhances the robustness of our findings and provides a deeper level of insight into the interplay between various parameters and their implications for practical applications.

We used 12 criteria to statistically analyze the efficiency of the exoskeleton assistance modes. These criteria are categorized into the following three groups. For statistical analyses, we used JMP 16.0 (SAS Inc., Cary, NC, USA).

### 3.1. Surface Electromyography (sEMG) Data

The processing of sEMG signals has become widely used during the last four decades to assess local muscle exhaustion [12]. In addition to processing this signal, we used the MuscleNET model [30] to estimate the joint torque from the sEMG signal and kinematic signals (IMU and BLDC motor angle). Six assessment measures are based on recorded sEMG signals.

**Measure 1**  A measure of sEMG amplitude employed in contemporary digital systems is the mean absolute value (MAV), also known as the average rectified value (ARV) (defined by Equation (1)), which is used as a time-domain fatigue evaluation method [12].
**Measure 2**  The power spectral density of an examined sEMG signal (Instantaneous median frequency (IMDF), defined in Equation (2)) changes toward lower frequencies during fatigue-inducing contractions [12,32].
**Measure 3**  Increase in median power spectral frequencies in comparison to the initial recording is another indicator of fatigue [33].
**Measure 4**  Equation (3) describes accumulated muscle activations during motion, and the square of muscle contractions is a fatigue metric frequently employed in neuromechanical models [34,35]. Here, filtered sEMG signals represent muscle contractions.
**Measure 5**  Each phase and participant had different sEMG channel amplitudes and patterns. We used the trained machine-learning model MuscleNET [30] to estimate shoulder elevation torque.
**Measure 6**  The instantaneous power of a human joint is the instantaneous torque estimated by MuscleNET [30] times the instantaneous angular velocity measured by the angular rotational sensor attached to the BLDC motor.

(1) $$MAV=\frac{1}{N}\sum_{i=1}^{N}\left|x_{i}\right|$$

(2) $$\int_{0}^{IMDF(t)}P(t,\omega )d\omega =\int_{IMDF(t)}^{\infty }P(t,\omega )d\omega =\frac{1}{2}\int_{0}^{\infty }P(t,\omega )d\omega$$

(3) $$FAT=\sum_{m}\int_{T}\sigma_{m}^{2}dt$$

where

MAV—the mean absolute value;
*x*—the amplitude of sEMG signal;
*N*—total number of signal points;
P(t,ω)—time-dependent power spectrum density of the sEMG signal;
ω—frequency of the signal;
IMDF—the IMDF;
σ—the muscle activations
*m*—the total number of sEMG channels;
FAT—the fatigue (**Measure 4** or **Measure 7**).

### 3.2. Kinematic Data and Inverse Dynamic Simulation

Here, we used a validated scalable musculoskeletal MapleSim model [36] to simulate and analyze the participants’ motion with the recorded kinematic data (joint angle, velocity, and acceleration).

**Measure 7**  By using the recorded joint kinematics and known external force/weight (e.g., exoskeleton assistance torque, the mass of the manipulated object, or the gravitational acceleration), we conducted an inverse dynamic simulation of the scalable musculoskeletal model (Equations (4) and (5)) [36] to estimate the activation of the muscle torque generator (MTG). Equation (3) was then used to calculate the computational fatigue.
**Measure 8**  Similar to **Measure 5**, the joint torque was calculated but from inverse dynamic simulation of the scalable musculoskeletal model (Equation (4) [36]).
**Measure 9**  Similar to **Measure 6**, the joint power was estimated with the inverse dynamic simulation of the scalable musculoskeletal model (Equation (4) [36]) and the instantaneous angular velocity sensed by the sensor of the BLDC motor.
**Measure 10**  The performance measurement of human motion is facilitated by the MMEE model. Kim and Roberts [37] combined thermodynamic rules with multibody system dynamics concepts to create a joint-space numerical model of MMEE. The energy model for zero co-contraction of MTG pairs is as shown in Equation (6).
**Measure 11**  The participants were expected to hold the kettlebell as long as feasible. The time that the shoulder elevation angle was more than 80% of the maximum angle (approximately $90^{\circ }$ as detailed in Section 2 and visualized in Figure 1a) was considered the load tolerance duration. This weight tolerance’s duration was quantitively compared after being recorded during different modes.

(4) $$\left[\begin{array}{cc} I_{2n\times 2n} & 0\\ 0 & M_{n\times n}\left(\theta_{n\times 1},\beta \right) \end{array}\right]\left[\begin{array}{c} {\dot{a}}_{2n\times 1}\\ {\dot{\omega }}_{n\times 1} \end{array}\right]=\left[\begin{array}{c} {{\dot{\tau }}_{u2a}\left(a_{2n\times 1},u_{2n\times 1},t,\beta \right)}_{2n\times 1}\\ F_{n\times 1}\left(\omega_{n\times 1},\theta_{n\times 1},\beta \right)+Q_{n\times 1} \end{array}\right]$$

(5) $$Q_{n\times 1}={\left[a^{+}\tau_{\omega }^{+}\left(\omega ,\beta \right)\tau_{\theta }^{+}\left(\theta ,\beta \right)\tau_{0}^{+}\left(\beta \right)+a^{-}\tau_{\omega }^{-}\left(\omega ,\beta \right)\tau_{\theta }^{-}\left(\theta ,\beta \right)\tau_{0}^{-}\left(\beta \right)+\tau_{p}\left(\theta ,\omega ,\beta \right)\right]}_{n\times 1}$$

(6) $$E=\int_{0}^{t_{max}}\left[{\dot{h}}_{M}{\left|\omega \right|}_{\left(max\right)}\left|\tau_{a}\right|+{\dot{h}}_{SL}\left|\tau_{a}\omega \right|+\tau_{a}\omega \right]dt$$

where

*n*—the number of independent coordinates = 20;
θ—the column matrix of all joint angles;
ω—the column matrix of all joint angular speed;
M—the mass matrix;
a—the muscle activation signal;
F—Coriolis, centrifugal, and gravitational effects;
Q—the applied joint torques, a column matrix containing $\tau_{h}\left(t\right)$ for all joints;
$\tau_{u2a}$—the excitation-to-activation signal ordinary differential equation (ODE) function;
u—the excitation signal;
$\tau_{\omega }$—the active torque–angular–velocity scaling function;
$\tau_{\theta }$—the active torque-position scaling function;
$\tau_{0}$—the peak isometric joint strength;
$\tau_{p}$—the passive torque function due to viscous damping and nonlinear stiffness;
$\tau_{a}$—the vector containing the active torques at the joints;
${}^{+}$—the positive direction of joint;
${}^{-}$—the negative direction of the joint;
β—the subject adjustment variables: sex, age, body mass, height, dominant side, and physical activity;
${\dot{h}}_{M}$—the dimensionless heat rate for activation and maintenance, determined to be 0.054;
${\dot{h}}_{SL}$—the dimensionless shortening lengthening heat rate, 0.283 for positive power, and 1.423 for negative power;
$\omega_{\left(max\right)}$—the maximum angular velocity over the entire motion.

### 3.3. Subjective Feedback

**Measure 12**  After conducting the exoskeleton performance test, participants engaged in a structured survey to gauge their experience comprehensively. This survey encompassed three key dimensions: participants’ self-reported fatigue levels throughout different exoskeleton phases (Table 1), the identification of specific areas of fatigue, and an assessment of comfort while wearing the exoskeleton on a scale of 1 to 10. The scale ranging from 1 to 10 for assessing comfort was a deliberate choice aimed at affording participants a broader spectrum of options to articulate their comfort perceptions. This scale was selected for its capacity to offer granularity in capturing the comfort levels expressed by participants in contrast to a binary scale that would provide only two options (e.g., comfortable or uncomfortable). Through this user feedback survey, we gained valuable insights into the interplay between exoskeleton assistance modes and user experiences. The survey’s structured approach allowed us to capture nuanced aspects of user interactions, offering a perspective that informs the practical usability and impact of the exoskeleton. This feedback enhances our understanding of how users respond to diverse assistance modes.

## 4. Results and Discussions

### 4.1. Quantitative Evaluation

**Figure 2:**
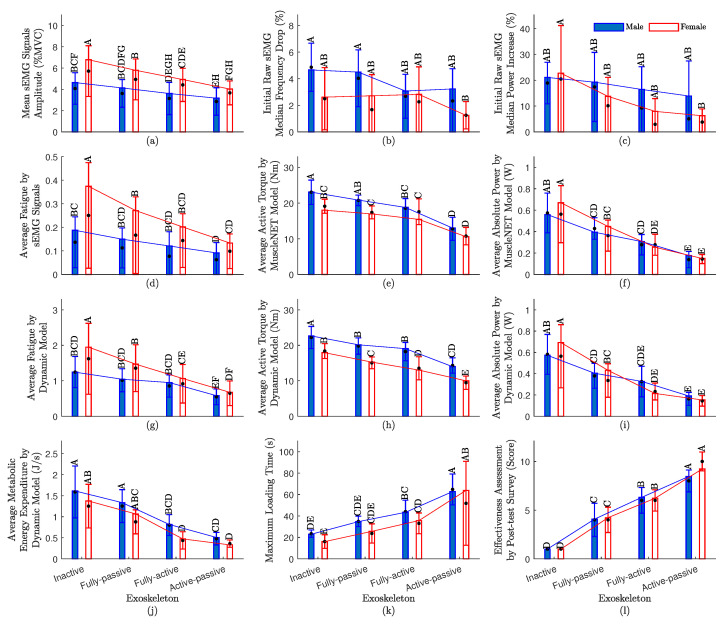
The sex impact and quantitative evaluation values for the four exoskeleton actuation types (IE, FP, FA, and AP). (**a**–**l**) are **Measure 1**–**Measure 12**; note that the median (.), the STD, and the Tukey honestly significant difference (Tukey HSD) letters are shown on top of all participants STDs for same-sex type (bar charts).

The results of the quantitative evaluation criteria are shown in Figure 2. The criteria for the average of all participants are shown as the solid bar, and the medians are shown as black dots; in addition, the male STD (blue line) and female STD (red line) from the mean value are shown on the bar charts.

To determine normality, the histograms of the data were visually examined for skewness and kurtosis. In Table 2, the criteria in Figure 2 were evaluated in terms of predicted root mean square of error (RMSE), *p*-value (a statistical metric that calculates the likelihood of obtaining the outcomes that were observed, supposing that the null hypothesis is correct), and R-squared (a statistical fit metric that quantifies the proportion of a dependent variable’s variance accounted for by the independent variables in a regression model). The general trend in Figure 2 and the following discussion are valid, since the *p*-value is less than 0.05 according to Table 2. The *p*-value is less than 0.001 considering the sex as a random variable (or a within-participant variable) since obviously each participant tested exoskeletons with one sex type and has not repeated it with another sex type.

To evaluate the effects of the fixed factors exoskeleton and exoskeleton condition times sex, we conducted Least-Squares Mean Differences and computed Tukey HSD test (using α=0.050 and Q=3.14619). For example, for metabolic energy expenditure in Figure 2j, the Tukey HSD is provided in Table 3. As can be seen, levels in Table 3 that are not connected by the same letter differ greatly, which means FP-female, FA-male, and AP-male are considered in the same category; the differences are minor but they are definitely better than IE and worse than AP-female.

**Figure 3:**
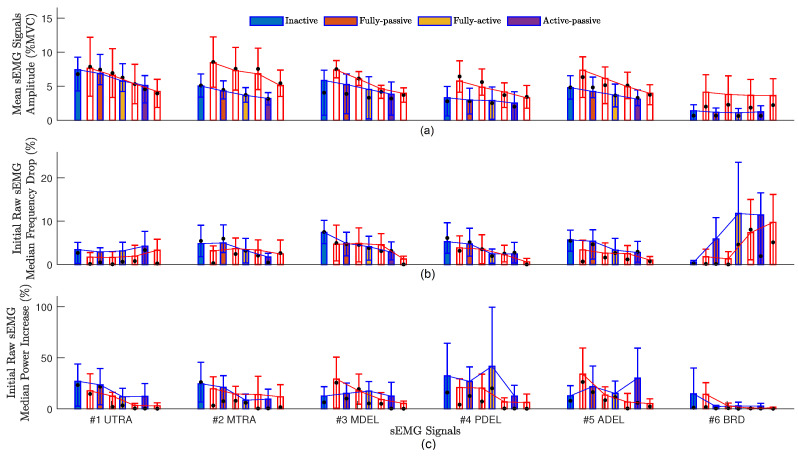
The muscle fatigue evaluations for the four exoskeleton actuation types (IE, FP, FA, and AP). (**a**–**c**) **Measure 1**–**Measure 3**. The male STD (blue line), the female STD (red line), and the median (black dot) are also shown on the average of all participants (bar charts).

Since the normalized RMSE by the mean value for **Measure 2** and **Measure 3** is high in Table 2, we provided more details (each sEMG channels) for these measures in Figure 3. For the majority of muscles, according to Figure 3, the following exoskeletons showed a decline in median frequency and a rise in median power from low to high: AP, FA, FP, and IE. This indicates that the AP exoskeleton reduced muscular fatigue. Nevertheless, a few muscles did not exhibit the same tendencies as the majority of the other muscles (Figure 3). For example, the sEMG signals from the BRD location showed no signs of fatigue. Since BRD is for elbow flexion/extension (EFE), these muscles and the joints they pass through were unaffected by the exoskeleton.

According to Figure 2, the most efficient exoskeleton types from powerful to weak are AP, FA, FP, and IE. According to Figure 2a–c,g,j, using the AP exoskeleton can decrease the fatigue level compared to using the FP exoskeleton.

As seen in Figure 2k, participants could lift the object for more time (until they became fatigued) by using the AP exoskeleton. The AP exoskeleton can be helpful for almost two times longer than the FP exoskeleton. In fact, the AP exoskeleton is adaptive to the task and can provide variable assistive torque instantly compared to the FP torque that can only provide fixed assistive torque at one specific angle, as mentioned in [38].

The participants scored the AP exoskeleton to be more effective than other exoskeletons (Figure 2l). Regarding the subjective feedback aspect, it is important to acknowledge that this section is primarily reliant on participants’ self-reported feelings and perceptions. Given our study’s sample size of 20 participants, we must be cautious when drawing concrete conclusions based on subjective assessments, particularly in areas involving mental and emotional aspects. The limitation stems from the relatively small sample size for such subjective evaluations, where larger participant groups would be more ideal to establish robust conclusions.

### 4.2. Sex Difference Perspective

From the sex differences perspective, a detailed evaluation of Figure 2a–d,g and Figure 3a–c reveals that females tend to experience more pronounced fatigue than their male counterparts when engaged in heavy lifting tasks. This observation underscores the importance of considering sex-specific ergonomic factors particularly in strenuous industrial settings that involve heavy lifting. To help mitigate the risk of MSDs among female workers and, consequently, enhance their overall well-being, the implementation of exoskeleton technology is strongly recommended.

It is noteworthy that the female participants in our study exhibited shorter stature compared to their male counterparts, resulting in relatively shorter arm lengths. This anatomical distinction has biomechanical implications, as shorter arms require less joint torque to lift objects of the same weight, which is a phenomenon supported by previous research [26,39]. Furthermore, shorter and lighter individuals tend to possess less muscle mass, contributing to produce less joint torque [26,39]. Consequently, females required less joint torque than males (Figure 2e,h).

Surprisingly, despite the lower joint torque requirements, females in our study demonstrated a significantly faster lifting pace compared to males. Actually, they required more shoulder adduction/abduction (SAA) power than males. This unique characteristic underscores the need for specialized considerations in exoskeleton design for females. Manufacturers should focus on optimizing exoskeleton joint stiffness to accommodate the increased speed of movement. Additionally, the control algorithm for such active exoskeletons should be tailored to effectively manage the specific biomechanical demands associated with rapid object manipulation.

It is important to note that in general, females tend to have a lower body mass and shorter stature compared to males. As depicted in Figure 2j, these anatomical differences contribute to a commensurately lower MMEE for females when compared to males. This characteristic highlights the significance of tailoring exoskeleton technology to accommodate diverse body types.

During the course of our study, several female participants expressed concerns regarding the fit of the exoskeleton belt. More precisely, regardless of the seated test condition, the exoskeleton belt was fastened over the female abdomen while a hip placement may have been preferred.

This feedback offers valuable insights for the future development of exoskeleton technology. Manufacturers can significantly benefit from considering the unique body shape and biomechanical requirements of female users when designing the kinematic fitting of exoskeleton attachments. By incorporating sex-specific design considerations, exoskeletons can be better optimized to enhance comfort and functionality for a broader range of users, ultimately promoting their adoption and effectiveness in various applications.

**Figure 4:**
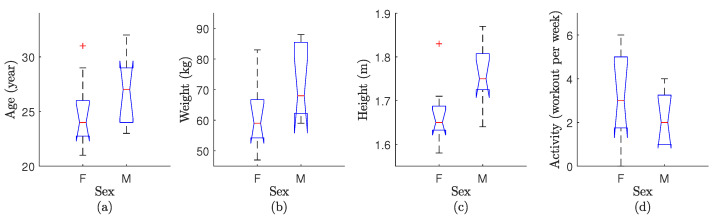
Depiction of a one-way analysis of variance conducted on the sample data of F: female versus M: male participants, with respect to their (**a**) age, (**b**) weight, (**c**) height, and (**d**) activity. Notably, the figure highlights an outlier through a red plus sign, while the median is represented by a red bar at the center. The minimum and maximum values are denoted by black minus signs at the bottom and top, respectively, and the 25th and 75th percentile values are enclosed within a blue bounding box.

It is important to remember that males and females have natural physical differences. These differences, like how muscles are distributed [40], the effects of hormones [41], and physical strength [26], can affect how people respond to the exoskeleton and how well they do in tasks [9]. Second, although this study did not select participants based on characteristics (e.g., height, weight, race, age, dominance side, hormonal levels, muscle distribution, or physical strength), the standard deviations of participants’ features (mean ± STD: 25±3.3years; 64±12.9kg mass; 1.74±0.09m height; 2.0±1.8 workout session per week; 18 right-handed and 2 left-handed) were within an acceptable range of deviation. To be more specific, employing a one-way analysis of variance, as illustrated in Figure 4, it is discerned that the median values for age, weight, and activity within one sex align closely with the main distribution of the corresponding attributes in the other sex. To be precise, the F-value (variation between sample means per variation within the samples) is 1.83, 3.51, and 0.96 for age, weight, and activity, respectively. It is noteworthy that despite females typically exhibiting shorter body height than males, their upper-limb length in proportion to body height is greater than that of males [42]. Furthermore, it is crucial to underscore that the test specifically entailed the lifting of a kettlebell with arms extended in a straight position, emphasizing the greater significance of upper-limb length over body height in this context. Third, the study’s primary goal was to selectively consider participant sex while treating all other participant features as random variables with minimal deviation. It is essential to note that adopting a selective approach, such as matching an overweight female with an underweight male to attain identical weight profiles, would compromise the consistency of the study cohort. Furthermore, it is worth noting that Hodson [9] and Rubio et al. [43] have previously deliberated on the concerns related to sex-based exoskeleton evaluation and advocated for a non-selective approach to participant recruitment.

### 4.3. Limitations

The present study rigorously assesses the performance of an AP shoulder exoskeleton, and the ensuing evaluation and discussion are specific to this particular exoskeleton model. While the participant cohort was substantial and well suited for objective statistical and scientific comparisons, it is important to acknowledge that the sample size may not have been sufficiently large to facilitate comprehensive subjective emotional comparisons, as elaborated upon in the survey section.

While, to the best of our knowledge, there exists only one AP shoulder exoskeleton worldwide, it is worth noting that a broader evaluation encompassing various AP shoulder exoskeleton models may yield a more comprehensive assessment.

Furthermore, in cases where a particular exoskeleton system can adjust torque in response to varying velocities, a comprehensive experiment involving different velocity settings, in addition to the examination of weight lifting scenarios, could provide valuable insights and a more holistic understanding of the system’s performance.

## 5. Conclusions

Robotic exoskeletons are becoming a more common tool for assisting industrial workers. The assisting source ranges from FP to FA. The effectiveness of four support modes were evaluated using the following types of assessment criteria: (I) sEMG-based, (II) kinematic-based, and (III) survey. The participants could hold the weighted object for nearly twice as long before becoming exhausted, indicating that the AP exoskeleton was superior to the FA and FP exoskeleton.

Future improvements should focus on proposing sex-specific controllers, accommodating anthropometric and joint demand differences, as well as the kinematic fitting of the wearable device for females.

## Figures and Tables

**Table 1:** The 6 steps of the test process, data collection, and exoskeleton calibration.

	Source		Tasks	
Phase Name	Passive	Active	Weight Lifting	Free Motion
Sensor Calibration				√
Data Gathering			√	√
IE			√	
FP	√		√	
FA		√	√	
AP	√	√	√	

**Table 2:** Statistical measures for different criteria: rmse, *p*-values, and R-squared.

Criteria	Figure 2	Signal		Model		Statistical Metric		
		Kinematic	sEMG	MuscleNET	Dynamic Model	Normalized RMSE by Mean Value (%)	Subject Wald *p*-Value	R-Squared
**Measure 1**	(a)		√			11.5	0.0021	0.95
**Measure 2**	(b)		√			52.7	0.0336	0.57
**Measure 3**	(c)		√			70.1	0.0167	0.60
**Measure 4**	(d)		√			29.7	0.0029	0.90
**Measure 5**	(e)	√	√	√		12.1	0.0307	0.91
**Measure 6**	(f)	√	√	√		30.9	0.0312	0.82
**Measure 7**	(g)	√			√	21.9	0.0033	0.90
**Measure 8**	(h)	√			√	8.0	0.0045	0.94
**Measure 9**	(i)	√			√	29.9	0.0115	0.82
**Measure 10**	(j)	√			√	28.2	0.0270	0.76
**Measure 11**	(k)	√				30.5	0.0244	0.78
**Measure 12**	(l)					18.1	0.0360	0.93

**Table 3:** A sample of Tukey HSD letters of metabolic energy expenditure for exoskeleton condition times sex factor. Levels not connected by the same letter are significantly different.

Category		Connections				Least Sq Mean
Exoskeleton Setup	Sex					
IE	Male	A				1.451
FP	Male	A				1.333
IE	Female	A	B			1.236
FP	Female	A	B	C		1.067
FA	Male		B	C	D	0.816
AP	Male		B	C	D	0.710
FA	Female				D	0.473
AP	Female				D	0.463

## Data Availability

The data generated and/or analyzed during the current study are not publicly available for legal/ethical reasons but are available from the corresponding author upon reasonable request.

## Abbreviations

The following abbreviations are used in this manuscript:

**ADEL**	anterior deltoid
**AP**	active–passive
**ARV**	average rectified value
**BLDC**	brushless direct current
**BRD**	brachioradialis
**EFE**	elbow flexion/extension
**FA**	fully active
**FP**	fully passive
**HITL**	human-in-the-loop
**IE**	inactive exoskeleton
**IMDF**	instantaneous median frequency
**IMU**	inertial measurement unit
**MAV**	mean absolute value
**MDEL**	middle deltoid
**MMEE**	muscle metabolic energy expenditure
**MSD**	musculoskeletal disease
**MTG**	muscle torque genera
**MTRA**	middle trapezius
**MuscleNET**	machine learning mapping electromyography to kinematic and dynamic biomechanical 407 variables
**MVIC**	maximum voluntary isometric contraction
**ODE**	ordinary differential equation
**PDEL**	posterior deltoid
**RMSE**	root mean square of error
**SAA**	shoulder adduction/abduction
**sEMG**	surface electromyography
**STD**	Standard deviation
**Tukey HSD**	Tukey honestly significant difference
**UTRA**	upper trapezius
